# Efficacy and safety of traditional Chinese medicine injections in the treatment of acute myocardial infarction: A protocol for systematic review and network meta-analysis

**DOI:** 10.1097/MD.0000000000021590

**Published:** 2020-08-07

**Authors:** Wei Zhao, Jun Li, Hengwen Chen, Qingjuan Wu, Yawen Deng, Yuqing Tan

**Affiliations:** a Guang ’anmen Hospital, China Academy of Traditional Chinese Medicine; b Beijing University of Chinese Medicine, Beijing, China.

**Keywords:** AMI, protocol, systematic review, traditional Chinese medicine injection

## Abstract

**Background:**

As one of the common cardiovascular diseases, acute myocardial infarction (AMI) is characterized by a high mortality rate, frequent complications, and a serious threat to human health and quality of life. Traditional Chinese medicine injection (TCMI) has been used clinically to treat AMI; however, there is no uniform standard for clinical treatment of AMI. The purpose of this study is to evaluate the efficacy and safety of different TCMI by using systematic review and network meta-analysis.

**Methods:**

According to the strategy, the authors will retrieve both 4 Chinese databases and 3 English databases by June 30, 2020. After a series of screening, randomized controlled trials will be included related to TCMI for AMI. Two researchers will use Aggregate Data Drug Information System and STATA 15.0 to analyze the data. Finally, the evidence grade of the results will be evaluated.

**Results:**

This study will provide a reliable evidence for the selection of TCMI therapies for AMI.

**Conclusion:**

The results of this study will provide references for evaluating the influence of different TCMI therapies for AMI, and provide decision-making references for clinical research.

**OSF registration number:**

DOI 10.17605/OSF.IO/FYGBT.

## 1 Introduction

Acute myocardial infarction (AMI) is caused by acute blockage of coronary arteries and interruption of blood flow, which causes continuous ischemia and hypoxia in myocardial cells.^[1]^ It is one of the diseases with the highest mortality globally and seriously threatens human health and quality of life.^[2]^ There are 5 types: Type 1 AMI caused by acute atherosclerotic thromboembolic coronary events; Type 2 AMI is caused by increased oxygen demand or decreased oxygen supply and may occur in patients with or without significant coronary artery disease; Type 3 AMI is only applicable to rare cases of death in patients with symptoms suggestive of myocardial ischemia. It can also be presumed to be newly occurred, ST-segment elevation, or new left bundle branch block. Type 4 and type 5 AMI are iatrogenic acute myocardial infarction associated with percutaneous coronary intervention (PCI) and coronary artery bypass grafting, respectively.^[3]^ However, in clinical practice, it is difficult to distinguish type 2 AMI from type 1 AMI, and it is also difficult to distinguish type 2 AMI from other myocardial injury or nonischemic diseases associated with elevated troponin, such as myocarditis, Takotsubo cardiomyopathy, or septic shock.^[4]^ Therefore, the prevalence of type 2 AMI varies greatly, ranging from 1.6% to 29.6%.^[5–7]^ The pathological factors of myocardial ischemia and infarction are various, including regional myocardial ischemia, global ischemia, myocardial shock, hibernating myocardium (chronic coronary insufficiency), myocardial necrosis, or fibrosis.^[8–11]^ Myocardial ischemia will seriously impair the systolic and diastolic functions of the heart,^[12]^ and patients with AMI are prone to irritability and fear, thus making sympathetic nerves excited, catechol amine level increased, myocardial load increased, and the disease worsened.^[13]^ Complications of AMI include various forms of hemodynamic insufficiency, such as acute heart failure, left ventricular remodeling, chronic heart failure, and cardiogenic shock.^[14,15]^

At present, the typical treatment of AMI patients is based on the main principles of opening infarcted blood vessels as soon as possible, promoting myocardial ischemia area reperfusion, saving dying myocardium, and actively managing complications.^[16]^ PCI operation is the preferred method in the treatment of AMI. However, the phenomenon of "no reflow (NR)" may be caused after surgery,^[17]^ the incidence ranged from 5% ∼ 50%,^[18,19]^ may lead to myocardial infarction area increases, affect heart function recovery, thereby increasing the forward end point events, lead to a significant portion of patients^[20]^ cannot obtain a satisfactory response to treatment. Therefore, the treatment of AMI is in urgent need of better treatment strategies.

Existing evidence shows that traditional Chinese medicine (TCM) has gained rich experience in the treatment of acute myocardial infarction, and the combination of TCM and Western medicine (WM) can further improve the prognosis of patients.^[21,22]^ At present, the main traditional Chinese medicine injection (TCMI) treatments for AMI include Shenmai injection, Shenfu injection, Xinmailong injection, Shengmai injection, Astragalus injection, etc.^[23]^ For example, shenmai injection can significantly reduce the ischemic myocardial area and increase the ejection fraction during early thrombolysis of AMI.^[24]^ Danhong injection (DHI) can reduce ST segment elevation myocardial infarction (STEMI) patients with high risk of reflow.^[25]^

At present, some studies have systematically evaluated the efficacy of TCMI in treating AMI. However, there is no network meta-analysis (NMA) on the differences between different types of TCMI in the treatment of AMI. The purpose of this study was to evaluate the efficacy and safety of different TCMI and to provide reference and evidence for clinical application.

## 2 Methods

### 2.1 Protocol and registration

The NMA protocol has been registered on the Open Science Framework platform (https://osf.io/fygbt), registration number: DOI 10.17605/ OSf.io /FYGBT. This protocol follows the Preferred Reporting Items for Systematic Reviews and Meta-Analyses Protocols guidelines.^[26]^

### 2.2 Ethics

Since NMA does not involve the collection of private information, this research does not require ethical approval.

### 2.3 Eligibility criteria

Five main factors of PICOS were used for the review: participant (P), intervention (I), comparator (C), outcome (O), and study design (S).

#### 2.3.1 Type of participant

All studies including patients with AMI diagnosed by any set of criteria were eligible for inclusion, such as 2012 ESC Guidelines on acute myocardial infarction (STEMI), Heart Disease and Stroke Statistics—2019 Update: A Report From the American Heart Association. Patients with other serious complications were not included, and the course and severity of the disease were approximately the same regardless of sex, age, nationality, or educational background.

#### 2.3.2 Type of interventions and comparators

Treatment group TCMI in combination with WM or taking TCMI alone, the control group didn’t intervene, placebo or WM. TCMI include Shenmai injection (SMI), Xinmai long injection (XMLI), Yiqi Fumai injection (YQFMI), Shenfu injection (SFI), DHI, Huangqi injection (HQI), Shenqi Fuzheng injection (SQFZI), etc.

#### 2.3.3 Type of outcomes

Primary outcomes

- Left ventricular ejection fraction (LVEF);
- N-terminal forebrain natriuretic peptide (NT-proBNP);
- Major cardiovascular adverse events (MACE).

Secondary outcomes

- ST segment regression degree;
- Venous thrombolysis rate;
- Serum inflammatory factors include high sensitivity C-reactive protein (hs-CRP), interleukin-6 (IL-6), and tumor necrosis factor-α (TNF-α);
- Adverse reaction (AR).

#### 2.3.4 Study design

This study is a systematic review with NMA of randomized controlled trials (RCTs) on the TCMIs for the AMI. All relevant RCTs using TCMIs for the AMI will be included. Quasi-RCTs, duplications, review documents, animal trails, clinical experience, and case reports will be excluded. Additionally, only Chinese and English literature will be searched for this study.

### 2.4 Literature retrieval strategy

Three English databases including Medline, The Cochrane Library, Embase and 4 Chinese databases were searched, including China Knowledge Infrastructure Database, China Biomedical Science, Chongqing VIP, and Wan-Fang. The time limit for literature retrieval is from the establishment of each database to 30 June 2020. The language is limited to English and Chinese. To obtain the potential nonelectronic literature, relevant magazines and medical journals will also be filtered for further search. In these databases, strict restrictions will be placed to exclude the types of studies that are not RCTs. Chinese search terms mainly include: “acute myocardial infarction;” English search words include “acute myocardial infarction,” “AMI,” “injection,” “Traditional Chinese medicine,” “Chinese herbal medicine.” Taking Medline as an example, the initial retrieval strategy is shown in Table 1 and will be adjusted according to the specific database.

**Table 1:**
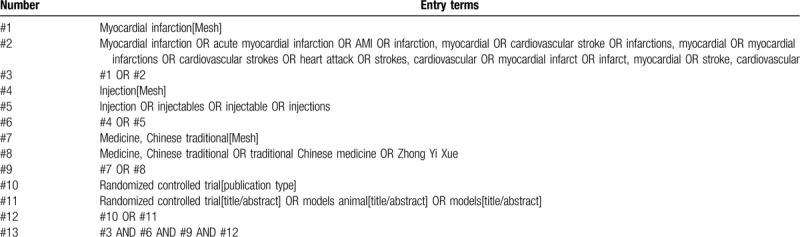
PubMed search strategy.

### 2.5 Literature selection and data extraction

**Figure 1:**
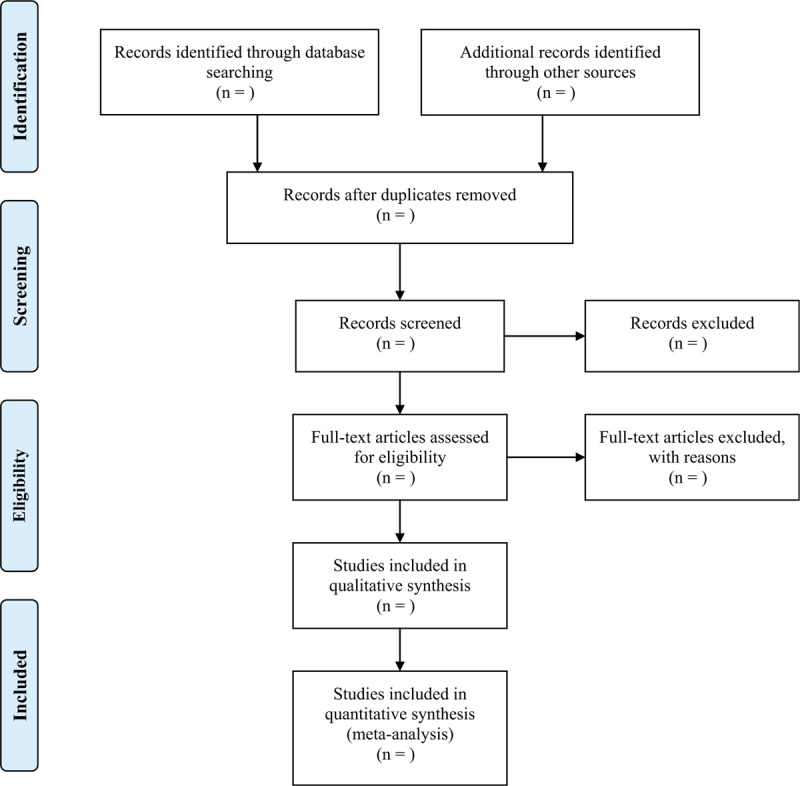
Flow chart of literature screening.

As shown in Figure 1, Wei Zhao and Yawen Deng will independently screen literatures according to inclusion and exclusion criteria: After importing the retrieved literature into EndNote X9.0 (Stanford, Connecticut, https://endnote.com), the duplicate literature was eliminated; Conduct a preliminary screening by reading the headline summary to exclude literature that does not meet the inclusion criteria; Reading the full text and making final selections; Data extraction using a predesigned data extraction table for the included literature and cross-checking the results; In case of disagreement, the third researcher Qingjuan Wu will be called upon to assist in judgment. Microsoft Excel (Redmond, Washington, https://www.microsoft.com/zh-cn) will be used to extract the data and collecting relevant information. Data extraction mainly included basic information of the literature (first author name, year of publication), basic information of study subjects (gender, average age, sample size, information of intervention and control group, intervention time, results, and follow-up time. The outcomes included LVEF, NT-proBNP, MACE, ST segment regression degree, venous thrombolysis rate, hs-CRP, IL-6, TNF-α, AR, etc. At the same time, the key factors of bias risk assessment are extracted. We will contact the corresponding authors for additional information if necessary.

### 2.6 Quality assessment/methodological quality of included studies

Methodological quality will be assessed based on the bias tool (ROB) in Cochrane Handbook 5.1.0. Two trained researchers Wei Zhao and Qingjuan Wu will independently evaluate the risk of bias of the included studies. In case of dispute, submit to corresponding author Jun Li for arbitration.

Cochrane bias risk assessment tool will be used to assess the risk of RCTs being included in NMA, 7 items are included:^[27]^ random Sequence Generation, allocation Concealment possibility; blinding of participants and personnel; blinding of outcome assessment; incomplete outcome data; selective reporting; other bias. Based on the above 7 entries, the judgment is divided into 3 levels: low risk of bias, high risk of bias, and unclear risk of bias.

### 2.7 Data synthesis and statistical methods

#### 2.7.1 Network meta-analysis

This study uses Aggregate Data Drug Information System (ADDIS) 1.16.8 for NMA,^[28]^ and uses Markov chain-Monte Carlo algorithm to make Bayesian inference. Iteration operations were performed according to the following preset model parameters: 4 chains were used for simulation analysis, with initial value of 2.5, a step size of 10, annealing times of 20,000 and 50,000 simulation iteration times. ADDIS software is used to draw network evidence diagrams of different outcome indicators, and odds ratio or standardized mean differences is used for statistical analysis, both with 95% credible intervals. According to the results of the NMA, rank probability plot of various TCMI therapies is generated and sorted by dominance, with Rank1 being the optimal sort.

#### 2.7.2 Statistical model selection

In this study, node-split model was used to analyze the consistency of data. When the statistical difference was compared directly and indirectly (*P* > .5), the consistency model was used for analysis. On the contrary, inconsistent model is adopted for analysis. If the consistency model is adopted, then the stability of the results is verified by the inconsistency model: When the inconsistency factors including 0, at the same time inconsistency standard deviation including 1 says the result of inconsistency model is more stable and reliable. At the same time, the preset parameters are used for iterative operation, and the convergence degree of iteration is judged by the potential scale reduced factor (PSRF). When the PSRF value is close to or equal to 1 (1≤PSRF≤1.05), the convergence is complete, indicating good stability of the model and reliable analysis results. If the PSRF is not in this range (1≤PSRF≤1.05), the iteration will continue manually until the PSRF value reaches the range standard.

#### 2.7.3 Heterogeneity test

Before the combination of effect size, the heterogeneity of the included literature is tested using STATA 15.0 software (Stata Corp, College Station, TX, https://www.stata.com). Evaluate the heterogeneity between studies through *I*^2^. When *I*^2^ > 50%, it indicates that the heterogeneity between studies is large, using a random effect model; when *I*^2^ < 50%, it indicates that the heterogeneity between studies is small or there is no difference qualitative, using a fixed effect model. When the heterogeneity is greater, the source of heterogeneity should be further sought.

#### 2.7.4 Sensitivity analysis

If necessary, sensitivity analysis will be used to assess the impact of the studies on the random effects model. After each study was excluded one by one, the data analysis was carried out again to determine the stability of the results. If there is no qualitative change in the combined effect shown in the results, the results are stable.

#### 2.7.5 Subgroup analysis

If there is clinical and methodological heterogeneity, we will conduct a subgroup analysis of the patient's age, onset of AMI, duration of treatment, or study quality.

#### 2.7.6 Small sample effect/publication bias

If 10 or more studies are included in the NMA, a comparison-adjusted funnel plot is developed using Stata to evaluate the presence of small sample effects or publication bias in the intervention network. If the plot is asymmetric and there is no inverted funnel shape, it indicates that there may be publication bias. The reasons may be related to the small sample size, allocation concealment, and insufficient implementation of blind method.

#### 2.7.7 Dealing with missing data

If the literature information is clearly incorrect or incomplete, we will contact the first author or the first author of the literature via email address. If no response is received, the document should be deleted.

#### 2.7.8 Evaluating the quality of the evidence

To grade evidence quality and understand the current situation of evidence rating thereby analyzing possible problems, the Grading of Recommendations Assessment, Development and Evaluation instrument will be used to assess the quality of evidence in the NMA.^[29]^ Based on bias, inconsistent, inaccurate, indirect, and the risk of publication bias 5 degradation factors, the quality classification for the 4 level of evidence: high, medium, low, and very low.

## 3 Discussions

As one of the common cardiovascular diseases, AMI has attracted extensive attention due to its rapid onset, rapid progression, high fatality rate, and poor prognosis. The incidence of AMI has been increasing year by year and is tending to be younger.^[30]^ PCI is the preferred method for acute myocardial infarction, and myocardial ischemia-reperfusion injury may occur after surgery, resulting in slow postoperative recovery of cardiac function in patients.^[31]^ In addition, PCI may promote the release of local inflammatory factors and vascular endothelial damage, leading to postoperative MACE.^[32]^ This study will discuss the efficacy and safety of TCMI in treating AMI from LVEF, hs-CRP, NT-proBNP, MACE, IL-6, and other aspects. As we all know, LVEF and NT-proBNP are important indicators to evaluate cardiac function, and the magnitude of NT-proBNP concentration can be applied to the early diagnosis of AMI and reflect the size of myocardial infarction area,^[33]^ suggesting cardiac function and quality of life of patients. hs-CRP is one of the factors that predict the risk of cardiovascular events,^[34]^ and its elevated level is positively correlated with cardiovascular risk.^[35]^ Studies have suggested that IL-6 level is closely related to long-term cardiovascular adverse events and mortality in patients with AMI.^[36,37]^ TCMI has recently been shown to play an important role in antimyocardial ischemia-reperfusion injury, possibly associated with increased expression of Bcl-2 and decreased expression of Bax and Caspase 3, as well as Akt/eNOS signaling.^[38,39]^

This NMA was designed to evaluate the efficacy and safety of TCMI in the treatment of AMI, and to provide a basis evidence for the clinical practice of Traditional Chinese medicine. In our study, a comprehensive analysis was conducted on RCT of all TCMI treatments for AMI, including SMI, XMLI, YQFMI, SFI, DHI, HQI, SQFZI, etc, so as to synthesize these evidences to make a comprehensive ranking of available TCMI treatments for AMI. Furthermore, this study has some potential limitations. For example, most of the studies published in Chinese journals did not have a strictly randomized design. In addition, due to the limited ability of the author, only English literature and Chinese literature were searched, which may lead to the risk of bias.

## Author contributions

**Conceptualization:** Wei Zhao, Yawen Deng, Hengwen Chen.

**Data curation:** Wei Zhao, Yawen Deng, Qingjuan Wu.

**Formal analysis:** Wei Zhao, Hengwen Chen

**Funding acquisition:** Jun Li, Hengwen Chen.

**Methodology:** Wei Zhao, Yawen Deng, Qingjuan Wu.

**Project administration:** Wei Zhao, Yawen Deng, Yuqing Tan.

**Writing – original draft:** Wei Zhao, Yawen Deng.

**Writing – review & editing:** Jun Li.
